# Gait Event Detection and Gait Parameter Estimation from a Single Waist-Worn IMU Sensor

**DOI:** 10.3390/s25206463

**Published:** 2025-10-19

**Authors:** Roland Stenger, Hawzhin Hozhabr Pour, Jonas Teich, Andreas Hein, Sebastian Fudickar

**Affiliations:** 1 Institute for Medical Informatics, University of Lübeck, 23562 Lübeck, Germany; roland.stenger@uni-luebeck.de (R.S.);; 2 Group Assistance Systems and Medical Device Technology, Department of Health Services Research, Carl von Ossietzky University Oldenburg, 26129 Oldenburg, Germany; 3 Section for Clinical Research IT (SKFIT), Institute of Medical Biometry and Statistics, University of Lübeck, 23562 Lübeck, Germany; 4 Fraunhofer Research Institution for Individualized and Cell-Based Medical Engineering (IMTE), 23562 Lübeck, Germany

**Keywords:** biomedical computing, biomedical signal processing, inertial sensors, wearable sensors, motion estimation, gait recognition, time series analysis, convolutional neural networks, machine learning

## Abstract

Changes in gait are associated with an increased risk of falling and may indicate the presence of movement disorders related to neurological diseases or age-related weakness. Continuous monitoring based on inertial measurement unit (IMU) sensor data can effectively estimate gait parameters that reflect changes in gait dynamics. Monitoring using a waist-level IMU sensor is particularly useful for assessing such data, as it can be conveniently worn as a sensor-integrated belt or observed through a smartphone application. Our work investigates the efficacy of estimating gait events and gait parameters based on data collected from a waist-worn IMU sensor. The results are compared to measurements obtained using a GAITRite^®^ system as reference. We evaluate two machine learning (ML)-based methods. Both ML methods are structured as sequence to sequence (Seq2Seq). The efficacy of both approaches in accurately determining gait events and parameters is assessed using a dataset comprising 17,643 recorded steps from 69 subjects, who performed a total of 3588 walks, each covering approximately 4 m. Results indicate that the Convolutional Neural Network (CNN)-based algorithm outperforms the long short-term memory (LSTM) method, achieving a detection accuracy of 98.94% for heel strikes (HS) and 98.65% for toe-offs (TO), with a mean error (ME) of 0.09 ± 4.69 cm in estimating step lengths.

## 1. Introduction

Gait analysis enables the identification of age-related musculoskeletal diseases and neurological movement disorders, while supporting the quantification of disease progression. For instance, gait patterns characterized by shuffled, small steps or lower gait velocities are associated with an increased risk of falling [1]. A previous study demonstrated a correlation, with confidence coefficients ranging from 0.595 to 0.798, between gait velocity, step and stride length, and the Berg Balance Scale [2]. Moreover, it has been shown that gait consistency and variability differ in individuals who fell within a 3-day study phase compared with healthy controls [3]. Another study indicated that gait velocity is associated with survival in the elderly population [4]. Changes in gait patterns can also manifest in the early stages of Parkinson’s disease (PD) [5,6]. Therefore, it is crucial to recognize such patterns as early as possible. Nonetheless, regular in-person screenings by specialists are often deemed impractical due to limited healthcare resources, patient discomfort, and lack of acceptance stemming from long travel distances, time constraints, or belief in their own health [7].

Gait analysis systems, such as floor-based gait tracking [8,9] and camera-based systems utilizing 3D or RGB cameras—with or without markers [10,11,12,13]—are valuable tools for estimating spatio-temporal gait parameters, including step length, velocity, and swing/stance time. However, these systems are typically confined to specific research settings due to their requirement for complex room setups. This includes sensor carpet implementation and advanced camera configurations, as well as extended subject preparation times, including marker placement. Continuous monitoring via camera-based systems in everyday life is also impractical due to privacy concerns that arise in private environments. Moreover, the installation of sensor carpets or camera-based systems in home environments incurs substantial costs, limiting monitoring capabilities to certain areas within the living space. Therefore, such ambient sensors are unsuitable for continuous gait analysis in everyday living.

IMU-based systems provide a cost-effective alternative. IMUs are widely available in consumer devices and have proven effective for gait analysis, typically when placed at the ankle or shank [14]. However, these placements can be inconvenient. In contrast, studies have shown that waist- or hip-mounted sensors, tested in populations with PD or Multiple Sclerosis (MS) [15,16], are both unobtrusive and comfortable, supporting their use for daily monitoring. Frequent monitoring supports clinically meaningful applications including fall-risk screening in older adults (e.g., discriminating high vs low fall risk using IMU features) [17]; early detection and monitoring in Parkinson’s disease, where IMU gait parameters correlate with disease status [18]; and rehabilitation tracking in MS or post-stroke patients, where IMU-derived gait features track recovery and asymmetries [19,20]. Bonanno et al. [21] systematically reviewed wearable-sensor fall-risk approaches in neurological populations, noting the presence of methodological heterogeneity and the importance of clinically grounded, standardized techniques.

In this work, we investigate gait event detection and parameter estimation using data from a single waist-worn IMU. We compare ML approaches including CNN and LSTM Seq2Seq models against GAITRite^®^ reference data. We further introduce an improved evaluation method using dynamic event-centered segmentation and demonstrate that waist-mounted IMUs can serve as a practical alternative to lower-limb sensors for accurate gait analysis. Because the waist offers a stable placement that is unobtrusive and easily implemented in a belt, it is especially suited for daily-life monitoring. While previous studies with waist-level sensors have predominantly focused on event timing, we extend this by demonstrating that a single waist-mounted IMU can reliably estimate temporal, spatial, and spatio-temporal gait parameters. This contribution broadens the scope of waist-worn IMUs and supports their use as a practical tool for everyday gait analysis.

## 2. Related Work

### 2.1. Use Cases

Two recent reviews have highlighted the diverse applications of IMU-based gait analysis. IMU-based gait analysis has been applied across diverse populations. A review by Mobbs et al. [22] reported that most studies focused on healthy individuals, while others examined older adults, patients with PD, stroke survivors, individuals with ataxia, Huntington’s disease, or lower-limb amputations. Similarly, Prasanth et al. [14] found that stroke-related impairments were the most common focus, followed by amputations, cerebral palsy, and PD. In both reviews, these conditions were identified retrospectively rather than being used as explicit searches.

### 2.2. Sensor Positions

The choice of sensor placement varies considerably across studies. Reviews show that foot and shank sensors remain the most common locations, often complemented by thigh or trunk placements in multi-sensor configurations [14]. Other studies report different distributions, with frequent use of wrist as well as waist, hip, and chest sensors [23]. Notably, when restricted to single-sensor setups, the waist emerges as the predominant location, selected in the majority of cases [22]. Evaluations highlight a sensor position trade-off; lower-limb placements (foot/shank) yield the most accurate detection of HS and TO events and better estimates of stance and swing times, whereas trunk/waist sensors are more practical to wear but can lead to reduced precision in these measures [24]. Cross-cohort tests further show that performance, particularly variability and asymmetry measures, is sensitive to sensor location and recording context (e.g., indoor vs. outdoor walking, healthy adults vs. PD), underscoring the importance of selecting the placement that best matches the target application [25].

### 2.3. Algorithms

Support vector machines have long been applied to gait analysis in the context of neurodegenerative diseases such as Alzheimer’s disease, PD, Multiple Sclerosis (MS), and Amyotrophic Lateral Sclerosis (ALS) [26]. More recently, deep learning methods such as CNNs and LSTMs have emerged, offering strong performance when sufficient training data are available, while instance-based and tree-based methods remain useful for smaller datasets.

Rule-based techniques, typically relying on thresholds or peak detection, are still widely used for gait event detection, particularly for HS and TO [14]. Their low computational complexity makes them suitable for real-time clinical applications, but they are generally limited to event detection and do not provide spatial or spatio-temporal parameters.

## 3. Materials and Methods

This section outlines the methodology used in the study, beginning with a description of the dataset collected using an IMU sensor and the GAITRite^®^ system. Two ML-based gait event detection algorithms are explained. The methodology also includes step length estimation and the calculation of additional gait parameters, which, in combination with the gait event detection approach, form a two-stage algorithm. Finally, the evaluation methods and performance metrics for the algorithms are presented. We chose CNN and LSTM Seq2Seq architectures because they are widely established baselines for time-series and wearable-sensor modeling, including human activity and gait from IMUs. Prior work shows CNN/LSTM as standard, high-performing choices in time-series reviews and in wearable sensing/HAR and gait estimation specifically [27,28,29,30,31,32,33].

### 3.1. Dataset

**Figure 1:**
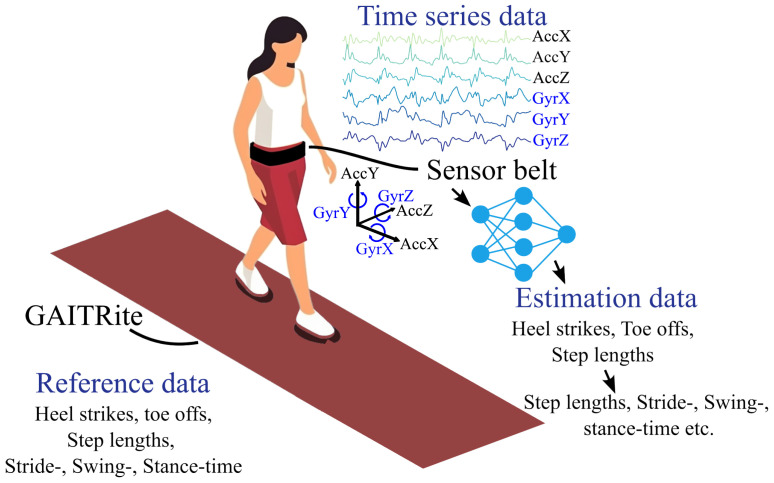
Schematic overview of the data that is used in this work. The reference data comes from GAITRite^®^, while the algorithms make estimations based on the measurements of the IMU sensor.

**Figure A1:**
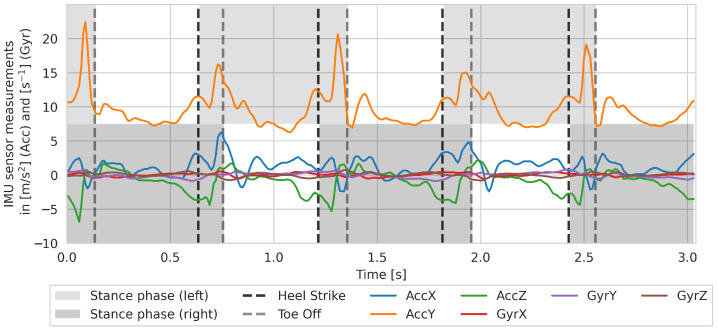
Exemplary time series of measurements from the sensor belt (IMU sensor) during a walk of a subject on a distance of 4 m. The y-axis indicates the vertical direction, which is why the data of AccY shows an increased acceleration.

The study dataset was collected at the University of Oldenburg using a single IMU sensor that was integrated into a waist-worn Humotion sensor belt, positioned between the L3 and L5 lumbar vertebrae [2,34,35]. This IMU sensor includes a triaxial accelerometer, gyroscope, and magnetometer, with data sampled at 100 Hz. The placement and orientation of the sensor were adjusted according to the hip circumference of each participant to ensure stability and uniformity, crucial for accurate gait recording (see Figure 1 for a schematic overview). Figure A1 shows the sensor data of a single walk with gait events (HS and TO) during a 4 m walk.

The GAITRite^®^ system, a validated tool for gait parameter estimation [36], was used as a reference, recording gait alongside the sensor belt. It records event-based measurements, capturing the exact time and location of foot contacts on its sensor grid, which are spaced at a minimum of 1.27 cm apart [9].

As summarized in Table A1, 69 subjects with ages ranging from 21 to 82 years are included in the dataset. The participants were healthy, with a median age of 57 years (standard deviation (SD) = 24; range: 21–82 years), of whom about 43% were female and 57% were male. Participants were instructed to walk continuously back and forth across the 4 m long GAITRite^®^ for 6 min. To capture a broader range of walking speeds, the walking test was conducted twice—once at a comfortable self-chosen (normal) pace and once at a self-chosen fast pace. Each walk was followed by short rest breaks. The walking speed conditions were not randomized across participants [34].

The raw triaxial accelerometer and gyroscope signals from the waist-worn IMU were normalized channel-wise to zero mean and unit variance. Magnetometer data were not considered, since the aim was to use a purely body-centered representation without incorporating global information. Step lengths were obtained from GAITRite^®^ as the Euclidean distance between consecutive HS positions. IMU and GAITRite streams were synchronized by estimating a constant time offset and shifting the IMU series accordingly. The raw IMU signals were used without additional filtering or transformation, apart from synchronization to GAITRite^®^ events and normalization, as described. Walks were excluded if either modality was missing, if the duration was shorter than 2.64 s (264 samples), or if GAITRite failed to detect at least one step. Missed steps were inferred when the preceding step time exceeded 150% of the median step time for that walk, which led to the removal of 36 walks (about 0.8%). Double steps were defined as step times shorter than 10% of the median and resulted in discarding 32 walks (about 0.7%), where the first of each double-step pair was removed. Data before the first HS and after the last HS were trimmed, and each IMU sample was assigned the GAITRite-derived step length of its enclosing HS interval, creating sample-aligned labels for Seq2Seq training.

### 3.2. Gait Event Detection

For comparison, two different gait event detection algorithms were implemented, designed as a Seq2Seq, based on convolutional or recurrent neural network RNN design, which are described in the following section. First, the common principles of both algorithms are introduced, followed by an explanation of the respective networks’ architecture and the training phase.

**Figure 2:**
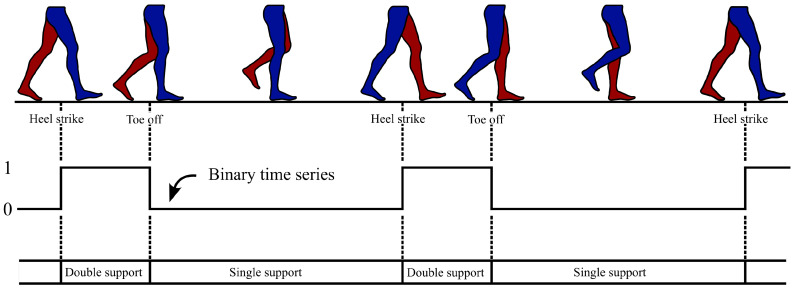
Representation of the human gait cycle through a binary time series. A value of 1 means that the step is in the double-support phase at the current time, while a value of 0 indicates single-support. This representation is used as a trainable label. The transition between the two phases mark HS or TO events.

The GAITRite^®^ data contains the timings of the HS and TO events per walk. Each walk $w_{ij}$ for subject *i* consists of HS timestamps $u_{ij}^{\mathrm{HS}}={\left(u_{1}^{\mathrm{HS}},u_{2}^{\mathrm{HS}},\ldots ,u_{k}^{\mathrm{HS}}\right)}_{ij}$ and TO timestamps $u_{ij}^{\mathrm{TO}}={\left(u_{1}^{\mathrm{TO}},u_{2}^{\mathrm{TO}},\ldots ,u_{l}^{\mathrm{TO}}\right)}_{ij}$. The number of gait events varies with each walk. To make this data available to the neural network as input, each walk is encoded by a binary time series. Each HS marks a new step and is the start of the double-support phase, meaning that two feet are touching the ground simultaneously. In contrast to that, the single-support phase is the phase where one foot touches the ground while the other is in the swing phase. Following this, the binary gait-support representation encodes for each time step whether it is a single- (0) or double-support phase (1). In this representation, gait events are identifiable at the transition points from 0 to 1 or vice versa, as depicted in Figure 2.

**Figure 3:**
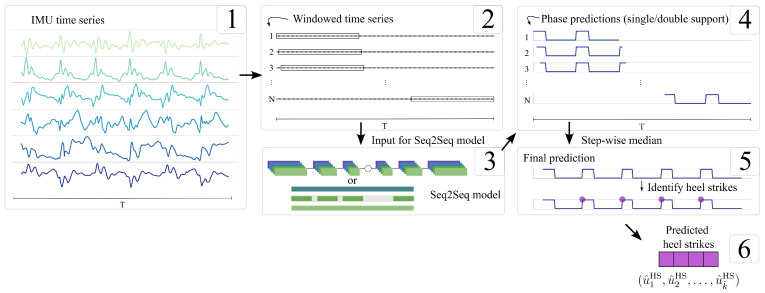
Overview of our windowing approach to sampling the time series (1), for same-length inputs (2), and for the CNN (3). To create a final estimation, overlapping fixed-length windows (264 time steps) with median filtering are used to derive event predictions (4–6).

Both neural networks process overlapping segments of the IMU signal. The six raw channels (three axes of acceleration and three axes of angular velocity) are divided into fixed-length windows N=264, extracted with a stride of one sample to ensure full coverage of the time series. For each window, the network outputs a sequence of the same length as the input segment, but with a single channel representing the estimated gait-support state at each time step (Figure 3). Because consecutive windows overlap, each time point is predicted multiple times.

Among the overlapping windows, the median of all binary gait-support estimations (potentially rounded) is calculated as the final output estimation. Therefore, for each input time series $X_{w_{ij}}\in R^{n_{wij}\times 6}$ with sequence length $n_{w_{ij}}\geq N\left(264\right)$, the network $N_{\mathrm{detector}}$ produces an output time series $N_{\mathrm{detector}}\left(X_{w_{ij}}\right)=\left({\hat{y}}_{1},{\hat{y}}_{2},\ldots ,{\hat{y}}_{n_{wij}}\right)$, where ${\hat{y}}_{t}\in \left\{0,1\right\}$ represents the type of gait-support phase. The transitions in that time series are used to identify the HS and TO events, resulting in two output vectors $\left({\hat{u}}_{1}^{\mathrm{HS}},{\hat{u}}_{2}^{\mathrm{HS}},\ldots ,{\hat{u}}_{k}^{\mathrm{HS}}\right)$ and $\left({\hat{u}}_{1}^{\mathrm{TO}},{\hat{u}}_{2}^{\mathrm{TO}},\ldots ,{\hat{u}}_{k}^{\mathrm{TO}}\right)$, where each value represents the estimated timestamp of either a HS or a TO of walk $w_{ij}$.

**Figure 4:**
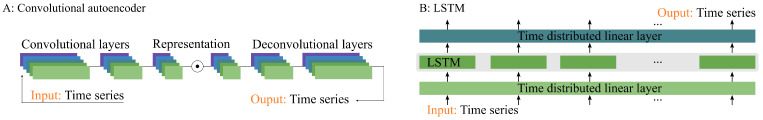
Two neural network architectures. (**A**) A Seq2Seq model using 1-dimensional convolutional and deconvolutional kernels, e.g., a convolutional autoencoder. Architecture (**B**) is based on an LSTM with time-distributed linear layers as encoder and decoder.

As visualized in Figure 4, the two neural network architectures are based on a CNN and an LSTM. The CNN consists of a three-layer 1D-CNN as an encoder, followed by a symmetrical set of deconvolutional layers. The first two convolutional layers consist of 64 kernels with ReLU activation, batch normalization, and dropout. The third convolutional layer employs 32 kernels with a ReLU activation. The convolutional layers use a stride of 2 and maintain a fixed padding of 1 with a kernel size of 3. Subsequent deconvolutional layers mirror the same structure, leading to a single, sigmoid-activated layer.

The LSTM is a 3-layer stacked formation, and takes a linear encoding sequence, where each step in the sequence is individually encoded into 64 features using the same linear layer (also known as a time-distributed layer). The LSTM’s output sequence is then decoded from 64 features to a single feature using another time-distributed linear layer, followed by a sigmoid-activated layer.

During training, time series of length 264 are sampled from the IMU data. A random offset is applied to each sample, and a sub-sequence of 264 length is then cut out. The AdamW optimizer [37] with a learning rate of 0.002, a weight decay of 0.01, and a batch size of 128 is used. The learning rate decreases by a factor of 2 as soon as the loss does not decrease over a span of 6 epochs. As a loss function, the Binary-Cross-Entropy loss for the gait event estimation is used. Leave-one-out cross-validation (LOOCV) is used to maximize the size of the training dataset. With LOOCV, the complete data of a single subject is withheld at a time and the training is executed with the rest. Only the unseen data of the left-out subject will be evaluated. The evaluation results are averaged over all folds. The training is stopped early if the loss does not decrease over a span of 24 epochs, or if the maximum number of 72 epochs has been completed.

### 3.3. Step Length Regression

**Figure 5:**
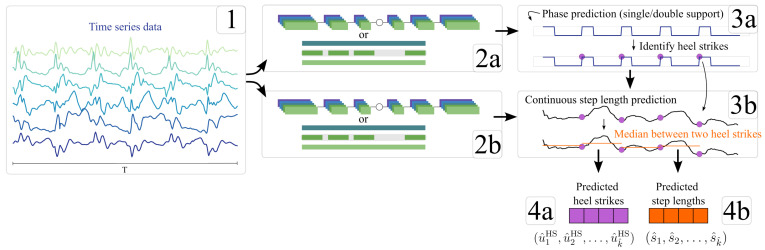
Combined HS detection and step length regression using a CNN or an LSTM as a Seq2Seq model with a windowing approach. The time series data from the sensor belt are fed as input (1) to the neural network (2a), which provides an estimation whether each time step is within a single- or double-support phase (3a). Based on this, the HS are identified as phase change from the single-support to the double-support phase (4a). The neural network for step length estimation uses the same input data (2b) and estimates for each time step a step length. The timestamps of the HS estimations are then used to average the section between neighboring events of the step length estimations (3b). Such averages are then used as the final step length estimation (4b).

In the following section, the algorithm for step length estimation for each consecutive HS event is described, which is visualized in Figure 5. The estimation of step lengths is based on the same neural network architecture (either CNN or LSTM) as the ML-based gait event detector. Only the last activation function (sigmoid) is replaced by the identity function.

The GAITRite^®^ data contains a step length annotation for each consecutive HS. As a preparatory step, the step lengths of each walk $w_{ij}$ are converted into a time series with the same length as the corresponding IMU data $X_{w_{ij}}$ of that walk. For each time step within the time span in between two consecutive HS, the same step length is assigned, given by the GAITRite^®^ data. The resulting time series $l_{w_{ij}}=\left(l_{1},l_{2},\ldots ,l_{n_{wij}}\right)$, with $n_{w_{ij}}$ as the total number of time steps within $X_{w_{ij}}$, therefore looks like a step function. $l_{w_{ij}}$ is to be estimated by the neural network, using the IMU data of a walk $X_{w_{ij}}$ as $N_{\mathrm{regressor}}\left(X_{w_{ij}}\right)={\hat{l}}_{w_{ij}}\to l_{w_{ij}}$.

Next, the HS event estimations are used for calculating the final step length estimations for each consecutive HS ${\left({\hat{u}}_{i}^{\mathrm{HS}},{\hat{u}}_{i+1}^{\mathrm{HS}}\right)}_{w_{ij}}$, with i∈{1,2,…,k−1}, where *k* is the number of estimated HS. The median of all estimations of the network $N_{\mathrm{regressor}}$ within the time spans between each consecutive HS is calculated. Additionally, a step length is estimated for the time span from time step 0 to the first estimated HS, resulting in a final estimation of step lengths ${\hat{s}}_{w_{ij}}=\left({\hat{s}}_{1},{\hat{s}}_{2},\ldots ,{\hat{s}}_{\hat{k}}\right)$. Note that $\hat{k}$, the number of predicted step lengths, may differ from the total number of step lengths, annotated by the GAITRite^®^.

For training, the same randomized sampling strategy as for ML-based gait event detection is applied. Furthermore, LOOCV, optimizer, learning rate, weight decay, batch size, learning rate scheduler, and early stopping strategy remain the same. The loss function used is Soft-DTW [38].

### 3.4. Additional Gait Parameter Estimation

The following gait parameters are calculated by using the HS and the respective step length estimations from the ML-based algorithm:

- Cadence: First, the inverse of the time between two consecutive HS is calculated, and the average is taken of all such values in each walk.
- Velocity: The velocity is computed by dividing each estimated step length by its corresponding step time.
- Step time: The step time is computed by subtracting the time of each estimated HS from the time of the previous HS.
- Stance time: The stance time is computed by subtracting the time of each TO from the time of the previous HS.
- Swing time: The swing time is computed by subtracting the time of each HS from the time of the previous TO.

These gait parameters are all evaluated walk-wise.

### 3.5. Evaluation

#### 3.5.1. Evaluation of Gait Event Detection

For the evaluation of gait event detection, each walk $w_{ij}$ is divided into segments. The segments are defined by using the midpoint between consecutive gait events (either HS or TO) as the start and the midpoint between the next pair of gait events as the end, using the annotations of HS and TO timestamps from the GAITRite^®^ ($t_{ij}^{\mathrm{HS}},t_{ij}^{\mathrm{TO}}$). The timestamp of the left boundary of the first segment is set to 0, and the right boundary of the last segment is set to $n_{w_{ij}}$. This segmentation ensures that each segment corresponds to exactly one reference gait event, allowing every estimated event in $u_{ij}$ to be uniquely assigned.

A predicted event ($P_{n}^{\mathrm{HS}}$ or $P_{n}^{\mathrm{TO}}$) is considered a true positive if it is the only detected event within the corresponding segment. Segments with multiple predictions are treated as over-estimations (false positives), while segments without any predictions are counted as false negatives. True negatives are not defined in this context, since by construction each segment is associated with one reference event. Based on this scheme, evaluation metrics such as accuracy, precision, recall, and F1-score are computed. In addition, the temporal precision of the detector is assessed by calculating the mean time difference between the estimated and reference events for all true positives.

This dynamic segmentation approach enables a meaningful assessment of temporal displacement between detected and reference events. Unlike fixed-window evaluation strategies, which can bias results through arbitrary window boundaries, the segmentation based on event midpoints provides an unbiased alignment to the reference data and captures the true temporal accuracy of the detectors.

#### 3.5.2. Evaluation of Step Length Regression

In the GAITRite^®^ reference data, each walk $w_{ij}$ has a varying amount of annotated step lengths $s_{w_{ij}}$, which correspond to the number of steps taken during the walk, which are defined by the number of HS occurring within $w_{ij}$. As a basis for evaluating step lengths, the steps must also be detected by the algorithm. When a HS $u_{n}^{\mathrm{HS}}\in u_{ij}^{\mathrm{HS}}$ is correctly detected (fulfilling the definition of a true positive according to Section 3.5.1), the corresponding step length estimation ${\hat{s}}_{n}\in {\hat{s}}_{w_{ij}}$ is used to calculate the error (mean error) with $s_{n}\in s_{w_{ij}}$.

## 4. Results

Prior to the evaluation, we investigated the influence of the window size parameter on the performance of the CNN-based gait event detection model. The window size determines the temporal span of input data considered for each prediction. We experimented with various window sizes, ranging from 8 to 496 time steps, with a step size of 16. In general, larger window sizes tend to produce better results. Beyond window sizes of 264 time steps, fewer walks meet the minimum length requirement, shrinking the training set. We found that this reduction in data outweighed any contextual gains from a longer window. Therefore, we selected a window size of 264 time steps for all subsequent evaluations, as it represented the best compromise between capturing sufficient gait event context and maximizing the utilization of our available data.

### 4.1. Evaluation of Gait Event Detection

The evaluation of the CNN- and LSTM-based gait event detectors is presented in Table 1 and Table 2. Both approaches achieved accuracy, precision, recall, and F1-scores consistently well above 98%. In terms of temporal precision, the CNN-based detector provided the lowest mean errors for HS and TO events (Table 2).

### 4.2. Evaluation of Step Length Regression and Additional Gait Parameters

Table 3 summarizes the performance of our step length regressions and the gait parameters derived from it. The CNN-based method outperforms the LSTM-based method for most gait parameters. Consequently, the CNN-based step length estimation is considered for further evaluation, as it demonstrated better performance overall compared to the LSTM-based approach. The mean bias was 0.09 cm (with SD 4.64 cm; 95% CI [0.026, 0.163] cm).

**Figure 6:**
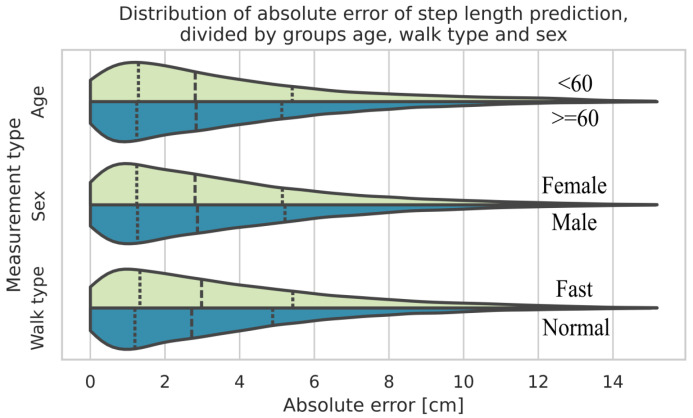
Distribution of absolute errors of estimated step lengths of the CNN-based approach, segmented according to age groups, sex, and types of walk.

When the data is grouped by walk type (normal and fast), age, and sex, the biggest difference between two of the respective groups results from the walk type, where fast walks show a bigger estimation error compared to normal walks. Subsequently, the percentage error between the walk types does not differ much (5.97% for normal walks compared to 6.28% for fast walks). Figure 6 presents a violin plot [39] that shows the kernel density estimations for each individual group in direct comparison with their counterparts. The dotted lines represent the median value and the quartiles, respectively.

The intraclass correlation coefficient (ICC) reflecting mean differences between subjects for the step length errors was 0.35. This indicates that about a third of the total variance in errors can be attributed to differences between subjects.

**Figure 7:**
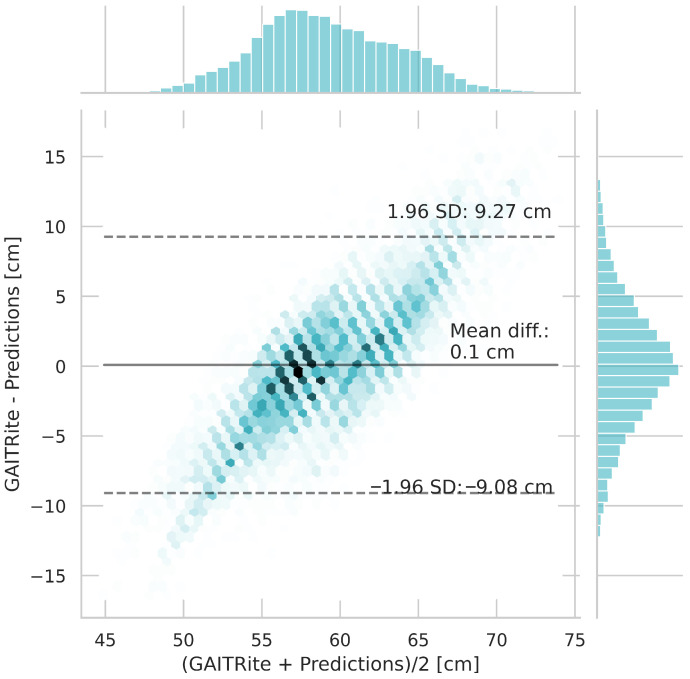
Bland–Altman plot of the step lengths estimations of the ML-based approach and reference values based on standard deviation (SD) and mean difference (Mean diff.).

Figure 7 shows the Bland–Altman [40] plot for the step length estimation of the ML-based algorithm. The mean difference is 0.09 cm with a 1.96 SD interval of [−9.08 cm, 9.27 cm].

**Figure A2:**
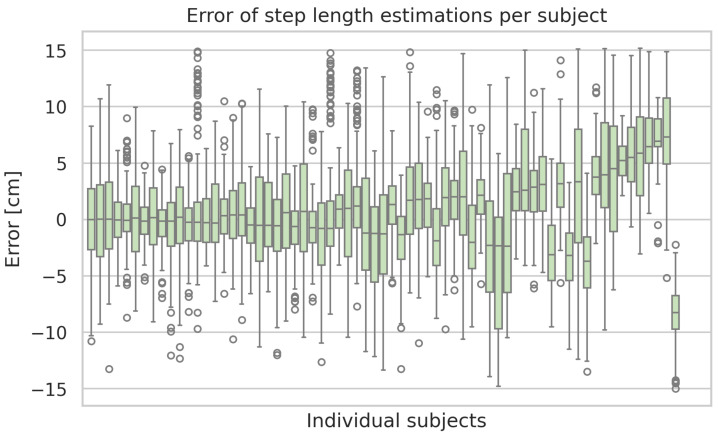
Box plots of the estimation errors of the ML-based approach for step lengths for each subject. Boxes show the interquartile range with medians, whiskers indicate the spread, and dots represent outliers.

If the error is calculated separately for each test subject, large differences become apparent as the error for some subjects exhibits a systematic bias. The mean error values on a single subject range from 0.01 cm to the biggest underestimate of −8.32 cm and +7.69 cm as the biggest overestimate on average. The standard deviation exhibits a minimum of 1.71 cm and a maximum of 6.47 cm for individual subjects. Figure A2 displays box plots of the step length error per subject. The same kind of plot for the remaining considered gait parameters is shown in the Appendix A.

## 5. Discussion

By comparison of two ML-based methods for detecting gait events, the study found that the CNN-based approach performed best overall.

Continuing with the results from the ML-based approach, the Bland–Altman plot showed a mean bias of 0.09 cm between the GAITRite^®^ system and the prediction model, indicating good overall agreement. Most differences (95%) fall within the limits of agreement (−9.08 cm to 9.27 cm). The random scatter of points around the mean difference line indicates no systematic error.

It is challenging to make direct comparisons between our results and related work due to the influence of various factors. These include differences in dataset size, reference systems, the course of the route (e.g., straight ahead, on a staircase, or going around a bend), evaluation metrics, or subject cohorts (e.g., healthy or diseased individuals, or other demographic characteristics). However, we do compare individual gait parameters by focusing on publications that implement gait analysis primarily on healthy subjects walking on a straight course. Table 4 and Table 5 show the results of previous studies in comparison with the results of our best-performing CNN-based approach. Compared to the studies, our algorithm can estimate respective gait parameters with a very low error, e.g., with respect to step length, our algorithm exhibits the lowest mean offset and standard deviation among the considered alternatives. Nevertheless, the algorithm from Verbiest et al. [41] performs particularly well regarding stride length, achieving a better mean and standard deviation compared to our approach for step length. For stance and swing time, our algorithm demonstrates mean and standard deviation errors that are intermediate compared to the other algorithms under consideration. With regard to step time, our algorithm has the second-smallest mean offset, while Vavasour et al. [24] have the smallest standard deviation (1 ms compared to ours, with 31 ms).

These differences can be partly attributed to variations in dataset characteristics and sensor placement across studies. Shank- and foot-mounted sensors capture more localized limb motion with higher acceleration amplitudes and less soft-tissue damping, which facilitates the precise identification of gait events and spatial parameters. In contrast, waist-mounted sensor data includes trunk motion and may be more sensitive to clothing movement and body composition, which may introduce additional variability. Moreover, study protocols differ in walk length, number of steps, and reference systems used, each of which can influence reported accuracy and comparability. Hence, some of the observed performance gaps likely reflect methodological and biomechanical differences rather than purely algorithmic limitations.

Our ML-based approach employs a windowing approach and requires a minimum time series length of 2.64 s in this instance. Consequently, the algorithm can be used in a streaming approach, whereby the results may be updated with each new time step in real time. In the initial phase, a minimum of 2.64 s of data history is required. The proposed algorithm is expandable to accommodate an arbitrary number of sensor channels. It is also possible to combine several sensors. In our case, we used one sensor, which resulted in six channels for the acceleration and angular velocity. To date, the algorithm has been developed for the purpose of detecting gait parameters during walking. During running and sprinting, the body experiences periods of no ground contact, complicating the detection of gait events using the binary representation of the gait cycle, which indicates single- and double-support phases. Nevertheless, an extension of the algorithm is planned to be developed to detect gait events during these gaits. This extension will take into account not only the single- and double-support phases but also a phase of no support. Currently, the algorithm is limited to identifying HS and TO events. Other gait events, such as heel-off and toe-on, are not detectable using our method.

While the proposed approach achieved high accuracy for gait event detection and strong agreement in step length estimation, performance on some derived temporal parameters was more modest, with only moderate correlations (0.55–0.57) for swing and stance times. This indicates that the method is best suited to the detection of gait events and step length, while secondary (derived) parameters may require further refinement. The moderate correlations for swing and stance times result from temporal inaccuracies at HS and TO detection that lead to error propagation in derived measures. Future work could address this by improving the event detection algorithm and incorporating more temporal context, for example, through transformer-based sequence models or temporal attention mechanisms.

Beyond this, our evaluation was limited to 69 healthy adults performing short, repeated 4 m walks in a laboratory setting, which restricts generalizability to pathological populations and real-world walking conditions. The 4 m length of the GAITRite^®^ runway also required frequent turning, which could in principle influence gait patterns and IMU signals. However, only straight-ahead walking segments, as instructed to the participants, were recorded for analysis. The GAITRite^®^ reference system itself has finite spatial resolution, which may introduce small errors. In addition, the algorithm requires 2.6 s of data history before producing first outputs, and accuracy may be affected by variability in waist sensor placement. Finally, the current event detection is restricted to HS and TO, while other gait events (e.g., heel-off, toe-on) remain undetected. The ICC of 0.35 indicates that about one-third of the variance in step length errors is explained by mean differences between subjects. This suggests that the model does not perform uniformly across participants, and an ICC closer to zero would be preferable for subject-invariant generalization.

## 6. Conclusions

Because waist-worn IMUs are unobtrusive, wearable, and cost-effective, they hold promise for use in continuous monitoring. While a significant amount of previous research has focused on determining gait parameters from IMU sensors placed on the foot or the shank, we introduced an algorithm for gait parameter estimation that achieves comparable accuracy to existing methods for foot and shank placements. Furthermore, the set of gait parameters derived from a single waist-worn sensor in our study addresses a gap in existing research. Notably, similar sensor setups have primarily concentrated on gait event detection and temporal parameters.

Our algorithm demonstrates convincing results when compared to existing works on shank-, foot-, or multi-sensor settings. These results are well within the range of corresponding common age- and disease-related variations. This could lead to earlier detection of gait abnormalities and improved management of gait-related disorders. Early detection of these conditions is crucial for timely intervention and improved patient outcomes. For instance, changes in gait patterns, such as shuffled, small steps, or lower gait velocities, are associated with increased fall risks and can be early indicators of neurological issues. The ability of our method to accurately estimate gait parameters from a waist-worn IMU sensor, being a minimally invasive approach, makes it particularly suitable for long-term continuous monitoring in everyday settings. Thus, we believe that the proposed system has the potential to impact the field of gait analysis by enabling continuous monitoring of gait patterns in everyday life settings.

Validating our algorithms on publicly accessible datasets would enhance comparability with other methods. However, to the best of our knowledge, there are currently no open-access datasets available that are comparable to ours regarding waist-worn IMU sensors with a GAITRite^®^ reference sensor. Nevertheless, we have maximized comparability by primarily comparing our approach to studies that utilize a similar dataset (i.e., healthy adults walking in a straight line). We intend to further evaluate the algorithm’s suitability for everyday living conditions in an upcoming study covering subjects in ecological gait conditions. In addition, potential applications include home monitoring and rehabilitation, where long-term gait tracking could inform timely interventions and therapy adjustments. Practical limitations such as battery life during continuous monitoring and variability in belt placement must be considered; these can be mitigated via power-aware operation and robust orientation handling (e.g., frequent self-calibration via instructions). Finally, integration into real-time wearable systems may enable applications in rehabilitation, fall-risk screening, or mobility monitoring.

## Figures and Tables

**Table 1:** Accuracy, precision, recall, and F1-score of the HS and TO detections. The better performance is marked in bold.

Metric	Method	HS	TO
Accuracy	LSTM	**99.03%**	98.54%
	CNN	98.94%	**98.65%**
Precision	LSTM	**99.05%**	**98.92%**
	CNN	98.93%	98.76%
Recall	LSTM	99.98%	99.61%
	CNN	**99.99%**	**99.80%**
F1-score	LSTM	**99.51%**	99.26%
	CNN	99.46%	**99.28%**

**Table 2:** Temporal deviation between estimated gait event timings and the true event timings, which is quantified by the mean absolute error (MAE) and mean error (ME) with standard deviation (SD). The better performance is marked in bold.

Metric	Method	MAE	ME ± SD
HS [ms]	LSTM	27.45	−2.34 ± 37.17
	CNN	**20.43**	**−1.45 ± 31.30**
TO [ms]	LSTM	26.89	−2.52 ± 40.82
	CNN	**21.75**	**−2.01 ± 36.56**

**Table 3:** Gait parameter estimation errors with mean error (ME), standard deviation (SD), and the Pearson correlation coefficient (CC) for the CNN. All results are highly significant (*p* ≪ 0.001).

Gait Parameter	ME ± SD	CC
Step length [cm]	0.09 ± 4.69	0.78
Cadence [1/min]	−0.266 ± 7.736	0.79
Velocity [m/s]	0.0047 ± 0.069	0.75
Stride time [ms]	−1.11 ± 43.48	0.79
Step time [ms]	0.88 ± 31.18	0.57
Swing time [ms]	1.01 ± 50.01	0.55
Stance time [ms]	0.88 ± 31.18	0.57

**Table 4:** Comparison of results (mean ± standard deviation) from previous studies with our ML-based approach—temporal gait parameters. For comparison, we considered only studies processing data from healthy subjects walking in a straight line.

Sensor Placement	Author	Stride Time [ms]	Step Time [ms]	Stance Time [ms]
Waist	Our	−1 ± 43	1 ± 31	1 ± 31
	[42]	6 ± 1	9 ± 3	13 ± 12
	[24]	0.2 ± 2	0.1 ± 1	-
	[43]	-	-	-
Shank	[42]	6 ± 2	9 ± 4	44 ± 13
	[31]	0 ± 30	-	-10 ± 40
	[33]	−21 ± 91	−8 ± 41	-
Feet	[31]	−10 ± 40	-	−10 ± 30
	[32]	0 ± 70	-	0 ± 70
Wrist	[43]	-	-	-
Multiple pos.	[44]	2 ± 20	2 ± 30	−8 ± 30
**Sensor Placement**	**Author**	**Swing Time [ms]**	**Cadence [1/min]**	
Waist	Our	1 ± 50	−0.27 ± 7.74	
	[42]	-	-	
	[24]	-	-	
	[43]	-	0.33 ± 1.9	
Shank	[42]	-	-	
	[31]	0 ± 30	0.6 ± 5.4	
	[33]	-	0.589 ± 1.144	
Feet	[31]	−10 ± 30	1.2 ± 6	
	[32]	0 ± 50	-	
Wrist	[43]	-	−0.07 ± 5.17	
Multiple pos.	[44]	10 ± 30	−0.296 ± 6.05	

**Table 5:** Comparison of results from previous studies with our ML-based approach (ML-Seq2Seq)–spatial (+Spatio-temporal) gait parameters. For comparison, we considered only studies processing data from healthy subjects, walking in a straight line.

Sensor Placement	Author	Step Length [cm]	Stride Length [cm]
Waist	Our	0.09 ± 4.69	-
Shank	[42]	-	-
	[31]	−0.6 ± 5.6	0.4 ± 9.7
	[33]	-	-
Foot/Feet	[31]	−1.7 ± 5.2	-3.0 ± 8.7
	[32]	-	−0.15 ± 6.09
	[41]	-	0.07 ± 4.3
Multiple pos.	[44]	0.6 ± 8	0.5 ± 7
**Sensor Placement**	**Author**	**Step Width [cm]**	**Velocity [m/s]**
Waist	Our	-	0.005 ± 0.069
Shank	[42]	-	-
	[31]	0.85 ± 4.6	-
	[33]	-	-
Foot/Feet	[31]	1.1 ± 5.1	-
	[32]	−0.09 ± 4.22	-
	[41]	-	-
Multiple pos.	[44]	0.8 ± 6	0.003 ± 0.05

**Figure A3:**
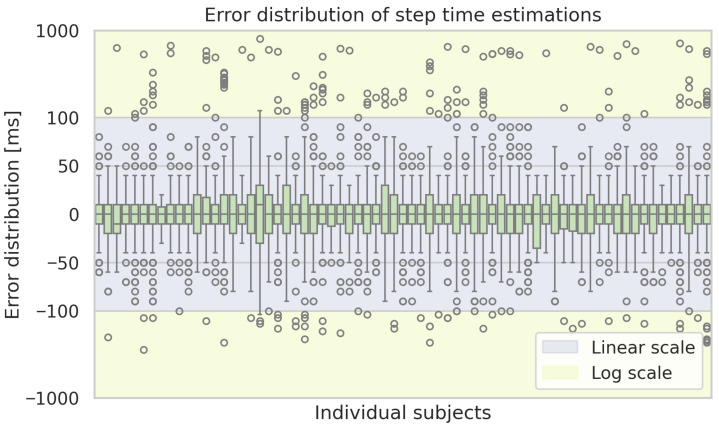
Box plots of the estimation errors of the ML-based approach of the step time estimations for each subject. Boxes show the interquartile range with medians, whiskers indicate the spread, and dots represent outliers.

**Figure A4:**
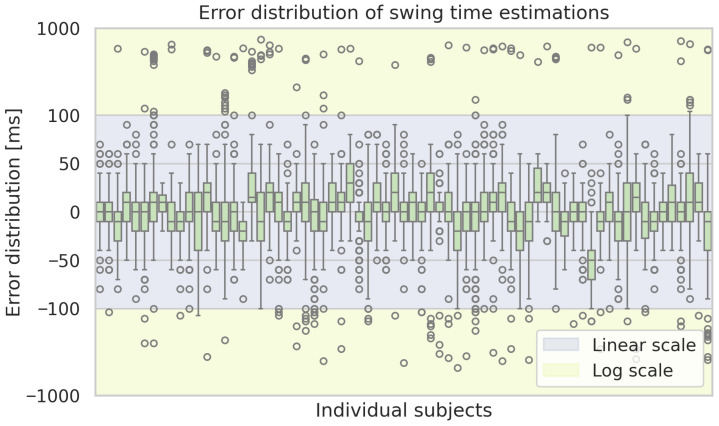
Box plots of the estimation errors of the ML-based approach of the swing time estimations for each subject. Boxes show the interquartile range with medians, whiskers indicate the spread, and dots represent outliers.

**Figure A5:**
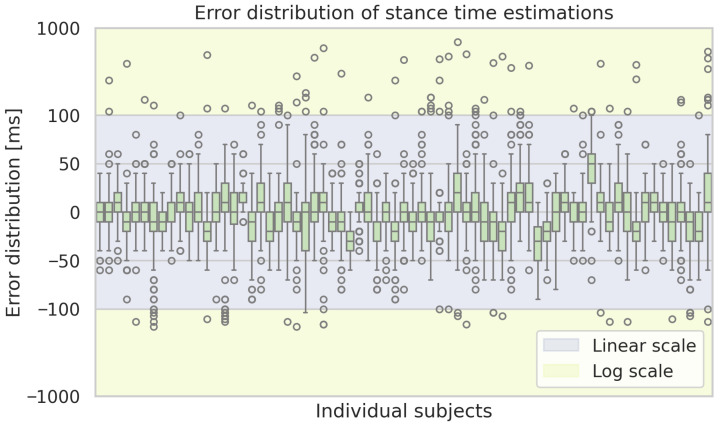
Box plots of the estimation errors of the ML-based approach of the stance time estimations for each subject. Boxes show the interquartile range with medians, whiskers indicate the spread, and dots represent outliers.

**Figure A6:**
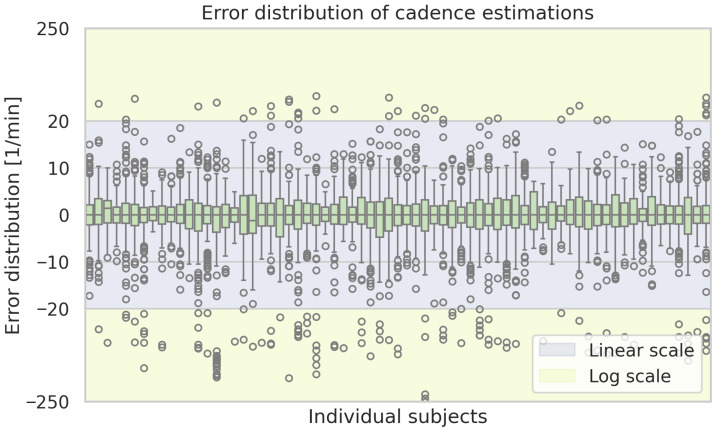
Box plots of the estimation errors of the ML-based approach of the cadence estimations for each subject. Boxes show the interquartile range with medians, whiskers indicate the spread, and dots represent outliers.

**Figure A7:**
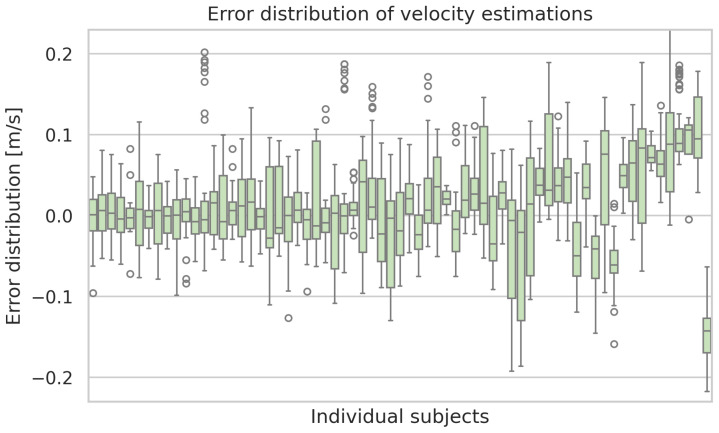
Box plots of the estimation errors of the ML-based approach of the velocity estimations for each subject. Boxes show the interquartile range with medians, whiskers indicate the spread, and dots represent outliers.

## Data Availability

The code for the algorithms presented in this study is available upon reasonable request from the corresponding author.

## Abbreviations

The following abbreviations are used in this manuscript:

IMU	Inertial Measurement Unit
HS	Heel Strike
TO	Toe Off
ML	Machine Learning
CNN	Convolutional Neural Network
LSTM	Long Short-Term Memory (network)
Seq2Seq	Sequence-to-Sequence (model)
LOOCV	Leave-One-Out Cross-Validation
SD	Standard Deviation
CC	Correlation Coefficient
MAE	Mean Absolute Error
ME	Mean Error
PD	Parkinson’s Disease
MS	Multiple Sclerosis
ALS	Amyotrophic Lateral Sclerosis

## Appendix A

**Table A1:** Statistics of the study cohort and the recorded data.

	Parameter	Mean (Std)	Min/Max
Subjects	N = 69		
	Height [cm]	173 (10)	151/196
	Weight [kg]	77 (13)	53/103
	Age [years]	57 (24)	21/82
	Walks per subject	66 (16)	28/92
Walks	N = 3588		
	Walk duration [ms]	3210 (725)	2000/7100
	Steps per walk	4.6 (1.2)	2/12
Steps	N = 17,643		
	Step length [cm]	59.22 (6.57)	36.85/85.09
	Stride time [ms]	574 (69)	410/2160
	Stance time [ms]	161 (37)	20/850
	Swing time [ms]	416 (55)	290/767
	Cadence [1/min]	105.7 (11.2)	81.0/146.0
	Speed [m/s]	0.86 (0.10)	0.63/1.20
